# Analyses of Antioxidative Properties of Selected Cyclitols and Their Mixtures with Flavanones and Glutathione

**DOI:** 10.3390/molecules27010158

**Published:** 2021-12-28

**Authors:** Joanna Płonka, Joanna Szablińska-Piernik, Bogusław Buszewski, Irena Baranowska, Lesław B. Lahuta

**Affiliations:** 1 Department of Inorganic Chemistry, Analytical Chemistry and Electrochemistry, Faculty of Chemistry, Silesian University of Technology, B. Krzywoustego 6, 44-100 Gliwice, Poland; Joanna.Plonka@polsl.pl (J.P.); irena.baranowska@polsl.pl (I.B.); 2 Department of Plant Physiology, Genetics and Biotechnology, University of Warmia and Mazury in Olsztyn, Oczapowskiego 1A/103A, 10-719 Olsztyn, Poland; Joanna.Szablinska@uwm.edu.pl; 3 Department of Environmental Chemistry and Bioanalytics, Faculty of Chemistry, Nicolaus Copernicus University, Gagarina 7, 87-100 Toruń, Poland; bbusz@chem.umk.pl; 4 Interdisciplinary Centre of Modern Technologies, Nicolaus Copernicus University, Wileńska 4, 87-100 Toruń, Poland

**Keywords:** antioxidant activity, radical-scavenging activity, liquid chromatography, cyclitols, flavonoids, glutathione

## Abstract

The conditions for determining the antioxidant properties of cyclitols (d-pinitol, l-quebrachitol, *myo*-, l-*chiro*-, and d-*chiro*-inositol), selected flavanones (hesperetin, naringenin, eriodictyol, and liquiritigenin) and glutathione by spectrophotometric methods—CUPRAC and with DPPH radical, and by a chromatographic method DPPH-UHPLC-UV, have been identified. Interactions of the tested compounds and their impact on the ox-red properties were investigated. The RSA (%) of the compounds tested was determined. Very low antioxidative properties of cyclitols, compared with flavanones and glutathione alone, were revealed. However, a significant increase in the determined antioxidative properties of glutathione by methyl-ether derivatives of cyclitols (d-pinitol and l-quebrachitol) was demonstrated for the first time. Thus, cyclitols seem to be a good candidate for creating drugs for the treatment of many diseases associated with reactive oxygen species (ROS) generation.

## 1. Introduction

In recent years, there has been a growing interest in inositols (cyclic six-carbon polyalcohols), including *myo*-inositol, present in all living organisms [1], and in their isomers (d-*chiro*-inositol, *scyllo*-inositol) and methyl derivatives (d-pinitol, l-quebrachitol), found in various wild and medicinal plants and crops [2,3,4].

*Myo*-Inositol, synthesized from glucose in three enzymatic steps, serves as a substrate for the synthesis of phosphatidylinositol, a major compound of cellular membranes, inositol high-energy pyrophosphates (implicated in phosphate and energy-sensing) and inositol phosphates (signaling molecules), including phytic acid (inositol hexakisphosphate) playing an important role in the storage/release of metal ions, as well as phosphate residues [5,6]. Moreover, *myo*-inositol in plants participates in the auxin transport/signaling, synthesis of cell wall compounds (hemicelluloses and pectin), and synthesis of galactinol and raffinose family oligosaccharides, compatible sugars [7,8]. *Myo*-Inositol and its isomers or methylated ethers (abundant in plants) are transported from source to sink tissues [9] and are accumulated to relatively high concentration under abiotic stresses, like drought or salinity, by participating in osmotic regulation and protecting the structure of macromolecules [10,11].

In humans, *myo*-inositol plays an important role in the processes of cell regulation, signal transduction, osmoregulation, and ion channel physiology, and is a component of the cell membrane [12]. *Myo*-inositol is involved in increasing the insulin sensitivity of various tissues. Together with d-*chiro*-inositol (a product of *myo*-inositol epimerization), regulates cellular glucose uptake and promotes glycogen synthesis [13]. Inositol deficiency may be involved in the pathogenesis of diseases, such as metabolic syndrome, spina bifida (a neural tube defect), polycystic ovary syndrome (PCOS), and diabetes [14,15]. Supplementation of the two inositol stereoisomers, d-*chiro*-inositol and *myo*-inositol is important to prevent these conditions [15]. Both are studied as metabolites with pro-health [12] and therapeutic properties, due to their insulin-sensitizing, anti-atherogenic, anti-inflammatory, anti-oxidative, and anti-cancer properties [12,14]. They are successfully applied in the treatment of PCOS and non-insulin-dependent diabetes mellitus [16,17]. The treatment with *myo*-inositol assists in the prevention of dyslipidemia, while combined therapy of *myo*-inositol and d-*chiro*-inositol improves the metabolic profile of obese PCOS patients, reducing the risk of cardiovascular disease [14]. The use of *myo*-inositol seems to be efficient for the treatment of depression, anxiety, and compulsive disorders [18,19]. Moreover, d-pinitol, indicating multifunctional properties (anti-diabetic, anti-cancer, hepatoprotective, antioxidant, anti-aging, and immunosuppressive), seems to be a good candidate for the treatment of various diseases [20,21,22]. Another methylated inositol—l-quebrachitol, chemically like d-pinitol (Figure S1), indicates gastroprotection, anti-platelet aggregation, anti-diabetic activity, and free-radical scavenging properties [23], and promotes osteoblastogenesis, while d-pinitol possesses inhibitory activity against osteoclastogenesis [24]. Thus, both cyclitols can be used in the treatment of osteoporosis. Although various mechanisms of cyclitols activity in the prevention/treatment of diseases are proposed [20,21,25], little is known about their antioxidant properties.

Some reports suggest that cyclitols act directly as scavengers of hydroxyl radicals [26] or indirectly, via enhancing antioxidative enzymes [22]. Such properties create an opportunity to use plant-derived cyclitols (mainly d-*chiro*-inositol and d-pinitol) in the treatment of some metabolic disorders, or plant food rich in cyclitols as a dietary supplement in the prevention of diseases [20,21,22]. However, analyses of the antioxidant properties of cyclitols with typical stable radicals, like DPPH (2,2-diphenyl-1-picrylhydrazyl), are rare [27]. Moreover, some reports indicate weak antioxidant activity of inositols [28] and their derivatives [29]. Thus, analyses of the antioxidant properties of the most important cyclitols (*myo*-inositol, d-pinitol, d-*chiro*-inositol, l-quebrachitol; Figure S1) seem important. Additionally, it could be hypothesized that cyclitols produce a synergistic effect with other antioxidants, like flavonoids [30], naturally occurring in plants and plant-derived food [31], or glutathione, an important antioxidant in both plants [32,33] and animals [34].

In plant tissues, apart from cyclitols, there are other compounds with antioxidant properties, for example, ascorbic acid (Vit C), glutathione, tocopherol (Vit E), carotenoids, anthocyanins, and various phenolic compounds, including polyphenols [35,36,37]. Among them, there are flavonoids with documented medicinal properties related to the regulation of ox-red processes in cells, including the maintenance of redox balance, disturbed by oxidative stress [38]. Disturbances in this balance are most often associated with the overproduction of reactive oxygen species (ROS). Oxidative stress damages the DNA and lipids change the structures and functions of proteins and carbohydrates and lower the concentration of intracellular ATP [38]. Many cardiovascular diseases are caused by oxidative modifications that disturb cellular homeostasis [39]. The multidirectional action of flavonoids is also manifested in their estrogenic, anti-inflammatory, antidiabetic, and cytostatic properties [40]. Oxidative stress plays a significant role in neurological diseases [41], including depression and neurodegenerative diseases [42,43]. Flavonoids are used as antidepressants, and their increasing use in medicine gives hope for the treatment of the so-called civilization diseases. Flavanones, which contain a stereogenic center and can therefore exist in the form of various enantiomers, are the least studied flavonoids in terms of antioxidant properties [44,45,46]. The flavanones that are the subject of this research include hesperetin, naringenin, eriodictyol, and liquiritigenin [47,48,49]. The spatial structure is significantly related to the possibility of fitting into an organism’s protein structures, for example, an enzyme. This is especially significant in the case of drugs because one of the isomers may have therapeutic properties while the other may be inactive or even have a negative effect (e.g., thalidomide, the *S*-enantiomer of which has proved to be teratogenic [50]).

Glutathione is an endogenous antioxidant that plays an important role in the functioning of living organisms. It is a tripeptide (γ-glutamyl-cysteinyl-glycine), a small intracellular thiol molecule considered to be a strong non-enzymatic antioxidant [32]. Its antioxidant properties rely on the restoration of thiol groups—SH in proteins that have been oxidized to disulfide -S-S- or sulfonic groups—SO_3_H. Owing to the free thiol group, GSH has the ability to reduce peroxides. It is also a coenzyme of some ox-red enzymes. Reduced glutathione (GSH) reacts with active oxygen species and thus protects protein thiol groups against irreversible inactivation [51]. Pereira et al. [52] noted the possibility of synergistic or antagonistic interactions between endogenous GSH antioxidant and exogenous antioxidants, for example, with some flavonoids. It has been pointed out that the presence of a catechol group in the B-ring of flavonoids is an essential condition for the synergism with GSH. The described studies were carried out in vitro, and it is expected that in in-vivo conditions the influence on the course of interactions will be additionally dependent on the bioavailability and biotransformation of the tested compounds, also in the presence of other metabolites, for example, cyclitols. In the present work, we show the results of our analyses of the antioxidant properties of selected cyclitols and their influence on the antioxidant properties of flavanones and glutathione.

## 2. Results and Discussion

### 2.1. Studies of Antioxidant Properties of Cyclitols by Spectrophotometric Methods

Due to the lack of data on the ox-red properties of cyclitols in the scientific literature, with the use of methods usually employed for this purpose, that is, the CUPRAC method or with the DPPH radical, systematic tests were carried out using both of these methods. In the preliminary study, using cyclitols at concentrations typical for an assay of antioxidative activity (in the order of μg/mL), any antioxidant activity of the tested cyclitols was detectable. Therefore, tests were carried out with the use of solutions with concentrations 1000-fold higher (in the order of mg/mL).

**Figure 1:**
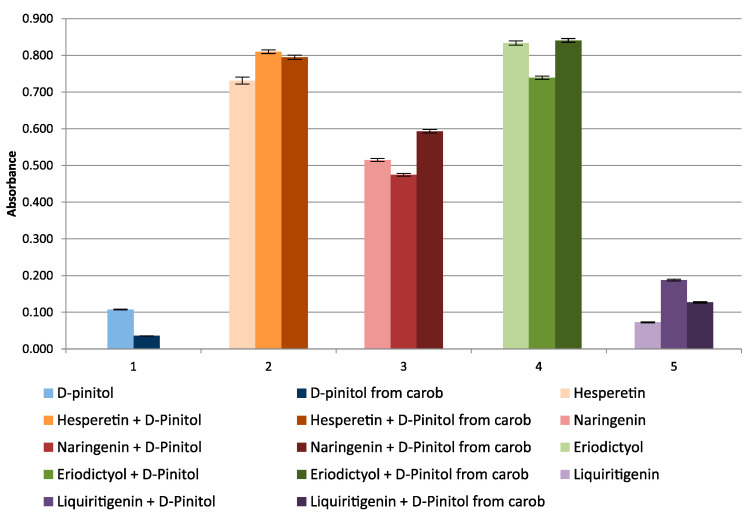
Absorbance change graph of d-pinitol (standard), d-pinitol from carob, flavanones and their mixtures with cyclitols.

**Figure 2:**
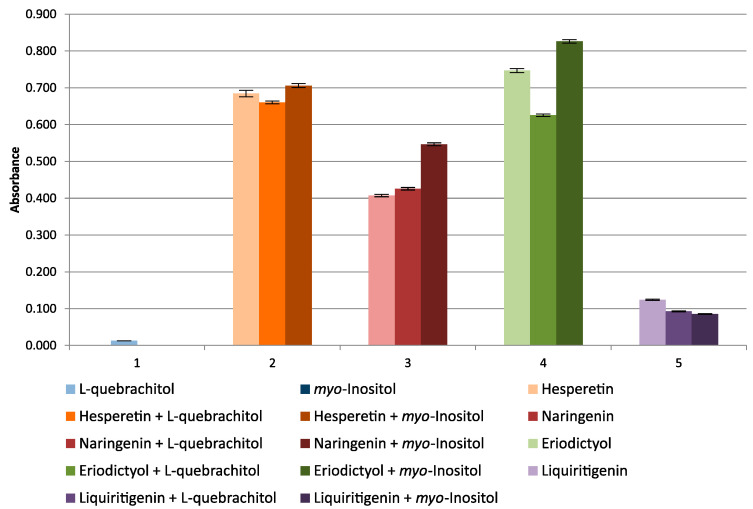
Absorbance change graph of l-quebrachitol, *myo*-inositol, flavanones and their mixtures with cyclitols.

In the CUPRAC method, a very weak interaction of d-pinitols (standard and obtained from carob), l-quebrachitol, and myo-inositol with Cu(II) ions was observed, and practically no reaction of the other inositols was tested. Tests were also carried out with the addition of known antioxidants, that is, flavanones, from the group of flavonoids (hesperetin, naringenin, eriodictyol, and liquiritigenin), at a concentration of μg/mL. The tested flavanones turned out to be strong antioxidants in the CUPRAC method, and d-pinitol (both standard and carob-derived) and myo-inositol added to the mixture of flavanones had only a slight effect on the ox-red properties of flavanones, causing little synergism or antagonism of action (Figure 1). The presented synergistic effect of d-pinitol from carob on the ox-red properties of flavanones can only be noticed at a high concentration of d-pinitol (in the order of mg/mL), at the concentration of flavanones from 5–15 µg/mL. Figure 2 shows the effects of l-quebrachitol and myo-inositol on the antioxidant properties of flavanones. Taking into account the very high concentrations of the cyclitols used, it should be assumed that their independent effect on the reduction of copper ions in in vivo conditions is unlikely.

**Figure 3:**
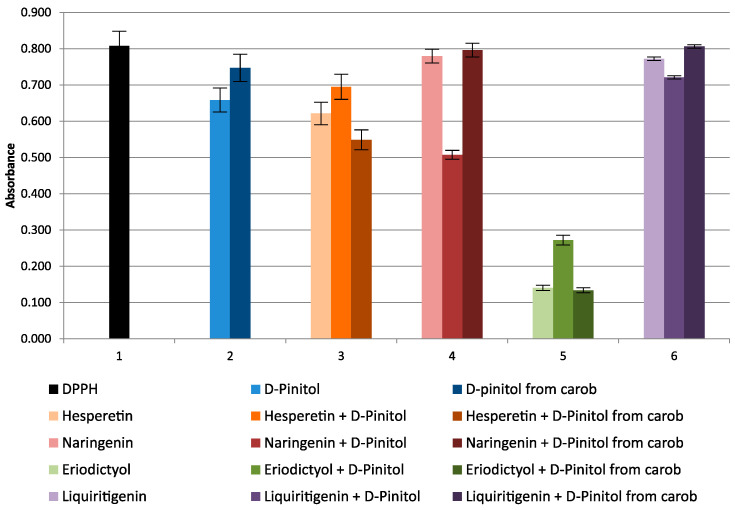
Changes in absorbance of d-pinitol (standard 11.7 mg/mL), d-pinitol from carob (11.7 mg/mL), flavanones (hesperetin, naringenin 16.7 μg/mL; liquiritigenin 20 μg/mL; eriodictyol 6.7 μg/mL), and mixtures of flavanones with cyclitols.

In the DPPH radical method, where the reaction is carried out in an alcoholic environment, only d-pinitol and l-quebrachitol were tested, as the remaining cyclitols precipitated in the reaction medium. Obviously, it was necessary to use solutions with a concentration of mg/mL due to the very poor antioxidant properties of the tested compounds. During the reaction, weak ox-red properties of the tested compounds in relation to the DPPH radical are observed—a slight lowering of the absorbance of the DPPH solution (Figure 3). It should be expected that the impurities present in d-pinitol isolated from carob (ca 5% of myo-inositol, traces of d-*chiro*-inositol, and glycerol, Figure S2) may affect its ox-red properties.

Studies of the reaction of d-pinitol with DPPH in the presence of flavanones were also carried out. Weak synergism or antagonism of the effect of d-pinitol on the ox-red properties of the tested flavanones was observed (Figure 3).

The reactions of DPPH with cyclitols in the presence of glutathione were conducted to explain the ox-red properties of cyclitols used in the treatment of various diseases. Results of our research on the antioxidant properties of cyclitols (Figure 1, Figure 2 and Figure 3) did not explain their positive effect on the treatment of diseases caused by oxidative stress. There was a suspicion that some endogenous compounds, present in every organism, may affect the results of the therapy as a result of synergism with cyclitols. Thus, it seemed natural to test glutathione, both separately and in mixtures with cyclitols and flavanones in reactions with the DPPH radical.

**Figure 4:**
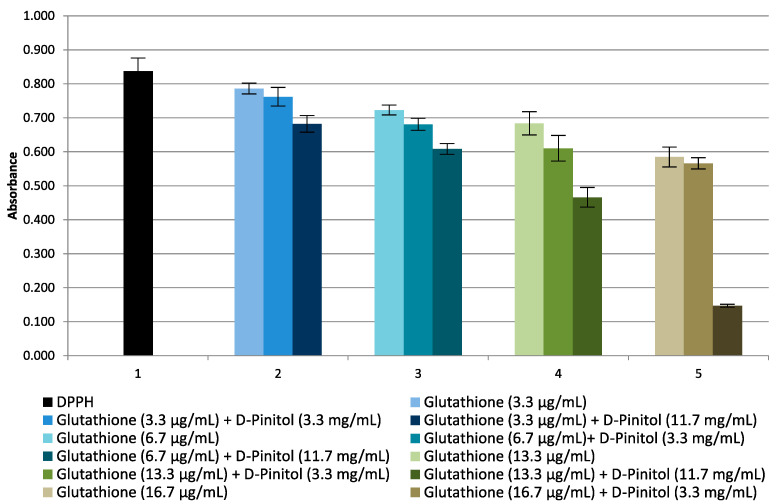
Changes in absorbance of glutathione and its mixture with d-pinitol (standard) at different concentration levels.

The influence of glutathione on the reaction with the DPPH radical was investigated and the quantitative dependence of the absorbance of the DPPH˙ solution on the amount of glutathione in the concentration range from 3 to 65 µg/mL was found. The effect of d-pinitol at concentrations of 3.3–11.7 mg/mL on reducing the absorbance of the DPPH˙ solution in the presence of 3.3–13.3 µg/mL of glutathione was investigated (Figure 4). There is significant synergy between the action of d-pinitol and glutathione. A similar synergy was found between the action of l-quebrachitol and glutathione (Figure S2). The influence of cyclitols on the antioxidant properties of glutathione depends on the type and/or origin of cyclitol.

Studies of the effect of glutathione on the antioxidant properties of flavanones in the DPPH radical method, not previously described in the literature, were also conducted. The studies were carried out with hesperetin, naringenin, eriodictyol, and liquiritigenin.

**Figure 5:**
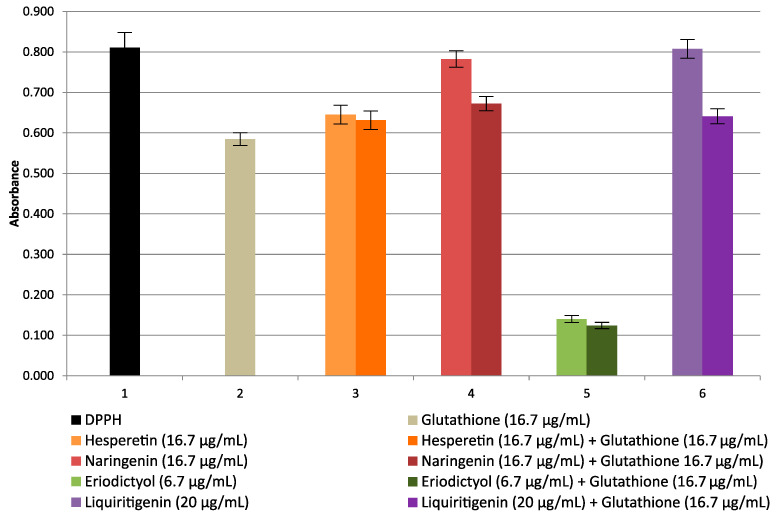
Changes in absorbance of glutathione (16.7 μg/mL), flavanones (hesperetin, naringenin 16.7 μg/mL; eriodictyol 6.7 μg/mL; liquiritigenin 20 μg/mL) and mixtures of individual flavanones with glutathione.

Figure 5 shows the changes in absorbance of the DPPH solution in the presence of glutathione, flavanones, and mixtures of glutathione with individual flavanones.

The effect of glutathione on the ox-red properties of hesperetin and eriodictyol with the DPPH radical is practically negligible. Weak antagonism is observed for reactions with naringenin and liquiritigenin. The differences are due to the influence of the structure of the flavanones studied on the formation of adducts with glutathione. Adducts formed at OH- groups of the ring B in flavanone and glutathione seem to be more important for the antioxidant capacity than adducts formed at OH- groups of the A ring.

A study of the effect of glutathione on the antioxidant properties of some flavonoids was conducted by Pereira [52], but it involved completely different flavonoids than the flavanones studied in this work. No studies have been conducted with cyclitols, which were the primary focus of this work.

### 2.2. Ultrafast UHPLC-UV Chromatographic Method for the Determination of Antioxidant Activities of Studied Cyclitols and Their Mixtures with Glutathione and Flavanones

Due to the very difficult solubility of most of the tested cyclitols in the reaction solution with the DPPH radical, for some of them, it was impossible to perform an analysis using the spectrophotometric method. In the spectrophotometric method, especially in view of poor ox-red properties of cyclitols, high concentration solutions had to be used, which in some cases resulted in precipitation of compounds during the measurements and made the measurements impossible. The advantage of chromatographic methods is the possibility of working with solutions of very low concentrations, so the determination of the antioxidant properties of cyclitols, flavanones, glutathione, and their mixtures with the DPPH radical by the UHPLC-UV was carried out. The author of the present study [53] has previously developed a UHPLC-UV procedure to study the ox-red properties of selected flavonoids (other than the flavanones studied in this work) without the presence of cyclitols or glutathione.

**Figure 6:**
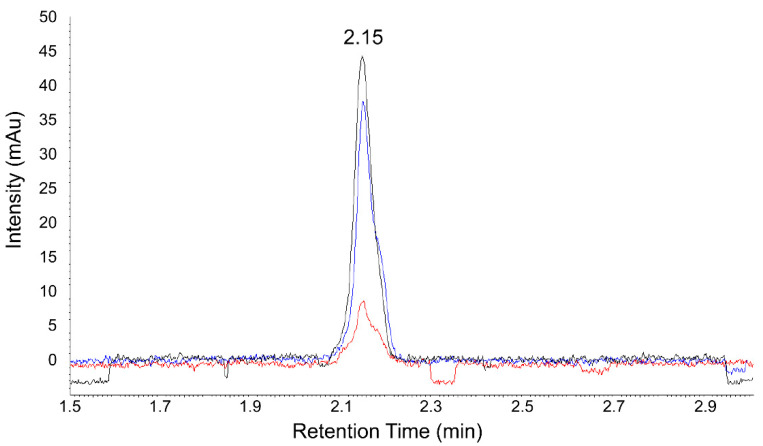
Example of a chromatogram of the DPPH radical signal (black line), DPPH radical signal after reaction with d-pinitol from carob (blue line), and DPPH radical signal after reaction with a mixture of d-pinitol from carob and glutathione (red line).

In the developed chromatographic method, the DPPH radical peak is observed on the chromatogram, with a retention time of T_r_ = 2.15 min at λ = 517 nm. During the reaction with the DPPH radical of cyclitols, flavanones, glutathione, and their mixtures, the peak area of DPPH˙ is reduced because of its reduction to DPPH-H, and these changes are proportional to the ox-red properties of the studied compounds. The peak area of DPPH radicals would decrease (as a result of color change from purple to yellow) when they encountered an antioxidant. Figure 6 shows an example of a chromatogram obtained for different mixtures of the examined compounds by the DPPH-UHPLC-UV method.

The difference in the reduction of the DPPH peak area (PA) between the blank sample and the sample of cyclitol, flavanone, or glutathione was used to calculate the percentage radical scavenging activity (% RSA) from the following Equation (1).

(1) $$\%\;\mathrm{RSA}\;=\frac{\left({\mathrm{PA}}_{\mathrm{blank}}-{\mathrm{PA}}_{\mathrm{sample}}\right)}{{\mathrm{PA}}_{\mathrm{blank}}}*100$$

Determination of %RSA values is a quantitative description of ox-red properties of the examined compounds. The present study, completely novel because of the examined cyclitols as well as flavanones and glutathione, was also carried out for mixtures of the analyzed compounds. The %RSA values determined in the present study for cyclitols, flavanones, and glutathione are shown in Table 1. In addition, the theoretical sum of RSA (%) of the mixture of cyclitol and antioxidant (Theoretical RSA% column) was calculated and the RSA for the mixture of cyclitol with particular antioxidants was determined experimentally (Experimental RSA% column). The arrows indicate the changes in RSA values.

The results show that the RSA% values for cyclitols are low (despite the use of high concentrations of the tested compounds), compared to those obtained for flavanones and glutathione, which confirms the very weak antioxidant properties of these cyclitols. Glutathione was found to be one of the strongest antioxidants among the examined compounds. Among the flavanones, eriodictyol has the strongest antioxidant properties and naringenin—the weakest. It should be noted that the RSA% values determined experimentally for specific mixtures differ significantly from those calculated for the sum of individual analytes. In mixtures of glutathione with cyclitols, the experimental values were in five cases higher or significantly higher than the theoretical ones (with d-pinitol from carob, with d-chiro-inositol and l-chiro-inositol), and practically unchanged with d -pinitol standard as well as with l-quebrachitol. It should be noted that the concentration of glutathione in the mixtures was 1000-fold lower than that of cyclitols. These are certainly very interesting data, given the constant presence of glutathione in living organisms, which will significantly affect the antioxidant properties of cyclitols used in medicine. In the case of mixtures of cyclitols with flavanones, antagonism occurred for most of the tested compounds, which may indicate the necessity of limiting flavonoid-rich products in the diet of patients, similarly to the treatment with some drugs for high blood pressure.

The concentration required to inhibit 50% of the radical scavenging effect (IC_50_) is also determined in works on the antioxidant activity of compounds. The IC_50_ index was determined from the results of a series of concentrations tested. A lower IC_50_ value corresponds to a larger scavenging activity. The values for selected compounds are presented in Table 2. IC_50_ values are given in concentration units, for example, μg/mL or mg/mL. The values obtained allow mutual comparisons of the ox-red properties of different compounds on the condition that measurements are carried out under the same conditions.

The data acquired from the determination of RSA and IC_50_ show that cyclitols are very weak antioxidants, but the ox-red properties increase significantly in the presence of glutathione. This is definitely important for the use of cyclitols as drugs. These data help to explain their efficacy and clarify the important role of human-derived glutathione as an essential factor involved in the therapy with these drugs.

## 3. Materials and Methods

### 3.1. Chemicals and Reagents

Cyclitols (*myo*-inositol, d- and l-*chiro*-inositol, l-quebrachitol and d-pinitol), flavanones (hesperetin, naringenin, eriodictyol, liquiritigenin), glutathione, DPPH (2,2-diphenyl-1-picrylhydrazyl radical), sugars (sucrose, trehalose, and monosaccharides—glucose, fructose, galactose), sugar alcohols (mannitol, xylitol, and ribitol), 1-(trimethylsilyl)imidazole, *O*-metoxyamine hydrochloride, *N*-methyl-*N*-(trimethylsilyl) trifluoroacetamide and pyridine were purchased from Sigma—Aldrich (St. Louis, MO, USA), and copper(II) chloride was purchased from Acros Organic (New Jersey, USA). Methanol, acetonitrile and trifluoroacetic acid (TFA, Merck, Darmstadt, Germany) were all HPLC grade. Analytical grade methanol, ethanol, and di-chloromethane were purchased from POCH S.A. (Gliwice, Poland). Doubly distilled water obtained from a laboratory purification system (Millipore, Milford, MA, USA) was used throughout the experiments.

### 3.2. Isolation and Purification of d-pinitol from Carob (Ceratonia siliqua L.)

#### 3.2.1. Extraction Procedure

d-Pinitol was isolated from carob molasses (Inkom AS, Istanbul, Turkey). For decolorizing and removal of major debris, 50 mL of molasses was dissolved in 100 mL of water and transferred to a Millipore vacuum filtration set packed with 200 mL of two-layer bed: 50 mL of silica gel (60 Å, 230–400 mesh, 40–63 μm particle size) and 150 mL upper layer of mixture (1:1, *v*/*v*) of activated charcoal (Sigma Aldrich, Saint Louis, MO, USA) and celite 545 (0.02–0.1 mm particle size, Merck). The double distilled water was used as the eluent. The colourless eluate (600 mL) was concentrated on a vacuum rotary evaporator (Heidolph, Hei-VAP Value, Schwabach, Germany) to 200 mL and subjected to fermentation with *Saccharomyces cerevisiae* Type II (1 g, Sigma Aldrich, Lot #BCBQ3003V) for the selective removal of interfering carbohydrates, according to [54], for 3–7 days (at 30 °C). The degradation of sugars was monitored daily, with GC-FID method [55]. Briefly, 1 mL of solution (taken into 1.5 mL Eppendorf tubes) was heated at 95 °C for 10 min and centrifuged at 14,000× *g* (at 4 °C for 20 min). A part of supernatant (400 μL) was purified on a micro spin filter (750 μL, 0.2 PVDF, Thermo Scientific, Waltham, MA, USA). The clear filtrate (10 μL) and 10 μL of xylitol (at 10 mg/mL, as an external standard) were transferred into a 2 mL glass chromatographic vial and concentrated in a speed vacuum evaporator to dryness. The dry residue was derivatized and analyzed by GC-FID at conditions described in the next section (Section 3.2.2). Sugars (sucrose, fructose, glucose, galactose, trehalose), mannitol, and cyclitols (*myo*-inositol, d-pinitol, and d-*chiro*-inositol) were identified by comparison of their retention times with those of original standards. The concentration of each soluble carbohydrate was calculated according to the appropriate regression coefficient (for each sugar at the concentration in the range of 10–200 μg/mL).

After the disappearance of sugars (sucrose, monosaccharides), yeasts were deactivated by heating for 10 min at 95 °C. Then, the solution was cooled, filtered through qualitative filter paper, centrifuged (3500× *g*, at 4 °C, 20 min), and concentrated to 25 mL under vacuum. The purification and fractionation of cyclitols were performed using ion exchange resins: firstly, the solution was loaded on a cation exchange resin column (length of 50 cm, ø 2.5 cm) packed with DOWEX 50Wx8 (200–400 mesh, Sigma Aldrich, Saint Louis, MO, USA). The eluate was concentrated and cyclitols were separated on an anion-exchange resin column (DOWEX 1x8, 200–400 mesh, Sigma Aldrich, Saint Louis, MO, USA), using double-distilled degassed water. Fractions containing mainly d-pinitol were combined, concentrated to 20 mL and d-pinitol was decanted with absolute ethanol (99.9%) at 4 °C for 24 h. The white precipitate was oven evaporated at 40 °C to dryness. The dry powder contained mainly d-pinitol, a several-fold lower amount of *myo*-inositol, and traces of d-*chiro*-inositol (Supplementary Material, Figures S3 and S4).

#### 3.2.2. GC-FID and GC-MS Analyses of d-pinitol

d-Pinitol purified from carob molasses as well as standard (from Sigma) was dissolved in water (10 mg/mL). Next, 5 μL of each was transferred into glass chromatographic vials and concentrated in a speed vacuum evaporator to dryness. Dry residues of both, pinitols and soluble carbohydrates (derived from fermented carob molasses—Section 3.2.1.) were derivatized with a mixture of 1-(trimethylsilyl)imidazole (TMSI) and pyridine (1:1, *v/v*). TMS-derivatives of cyclitols and soluble carbohydrates were analyzed by GC-FID method on a gas chromatograph GC2010Plus (Shimadzu, Kyoto, Japan) with a capillary column ZEBRON ZB-1 (15 m length, 0.25 mm diameter, and 0.1 μm film thickness, Phenomenex, USA). The injector temperature was 325 °C and the initial column oven temperature was 150 °C. Helium was used as carrier gas (at a flow rate of 1.18 mL/min). After the sample injection (1 μL, in a split ratio 10:1), the oven temperature (150 °C) was ramped to 200 °C at a rate of 20 °C/min, to 300 °C at 30 °C/min and to 335 °C at 20 °C/min. The final temperature was held for 10.42 min and the total time of analysis was 18 min. The detector was maintained at 350 °C.

GC-MS analysis was performed to compare the mass spectra of d-pinitol obtained from carob with those of the original standard. The GC-MS analytical method has been previously described in detail [56]. Briefly, the dry residues were derivatized in two steps. Firstly, 40 μL of *O*-metoxyamine hydrochloride (20 mg in 1 mL of pyridine) was added and samples were heated at 37 °C for 75 min (with continuous shaking at 500 rpm). Then, 160 μL of the mixture of *N*-methyl-*N*-(trimethylsilyl) trifluoroacetamide with pyridine (1:1, *v*/*v*) was added and heated at 70 °C for 30 min (according to the method described by Lisec et al. [57]. The TMS-derivatives of cyclitols were analyzed using GC-2010 coupled with a quadrupole mass spectrometry (MS) analyzer (GCMS-QP2010 Plus, Shimadzu, Kyoto, Japan), identified by comparison of the retention time and mass spectra of original standards (derived from Sigma-Aldrich, USA) and from the NIST 05 library (National Institute of Standards).

### 3.3. The CUPRAC Method Applied to Study the Ox-Red Activity of Cyclitols and Their Mixtures with Flavanones

Aqueous solutions of cyclitols with a starting concentration of 100 mg/mL and methanolic solutions of flavonoids with a starting concentration of 1 mg/mL were prepared. Solutions of copper(II) chloride (0.01 M Cu^2+^) in water, neocuproine (7.5 mM) in ethanol, and ammonium acetate (1 M) in water were prepared. Absorbance measurements of copper(II) and neocuproine solutions, after addition of the cyclitols under study, followed by cyclitols in a mixture with flavonoids and separately flavonoids alone, were carried out at a light wavelength of λ = 450 nm using a Unicam SP 1700 spectrophotometer. All measurements were performed in triplicate.

### 3.4. Spectrophotometrically DPPH-UV Radical Scavenging Assay

A total volume of 100 μL aliquot of each standard of cyclitols, flavonoids, or glutathione or a mixture of these compounds (at different concentrations, depending on the compound) was mixed with 350 μL of 200 μM DPPH in methanol and brought with methanol to a final volume of 1.0 mL. After 30 min, absorbance was measured at λ = 517 nm against a blank, containing all reagents except the tested samples. Assays were performed in triplicate. Under the described conditions, it was not possible to carry out tests with *myo*-inositol, l-*chiro*-inositol, and d-*chiro*-inositol because they precipitated out of the solutions during the reaction due to their very low solubility in methanol.

### 3.5. DPPH-UHPLC-UV Radical Scavenging Assay

The antioxidant activity was assessed with a UHPLC-UV analysis. Before the DPPH –UHPLC-UV analysis, the samples were prepared in the same way as described above. After the reaction, the samples were injected for UHPLC-UV analysis. The UHPLC system (Merck Hitachi, Germany) consisted of a pump (Model L-2160U), UV detector (Model L-2400U), autosampler (Model L-2200), a temperature-controlled column compartment (Model L-2350U), and a degasser module. The EZ Chrom Elite System Manager was used for data acquisition and analysis.

Chromatographic analysis was conducted according to the following procedure—column: Synergi C18 Fusion-RP (50 × 2.0 mm, 4 μm, Phenomenex), mobile phase: (A) 0.05% TFA in water and (B) acetonitrile (linear gradient from 30% B to 80% B in 2 min; then changed to 30% B immediately and held at 30% B for 2 min to equilibrate the column); temperature: 25 °C, injection volume 2 μL, detection UV λ = 517 nm (monitored DPPH˙ peak). The retention time Tr of the DPPH signal was 2.15 min [53].

## 4. Conclusions

Extensive, completely novel studies of the antioxidant properties of a wide spectrum of compounds—cyclitols, flavanones, and glutathione, separately and their mixtures (in different concentration ranges), by the CUPRAC, DPPH-radical, and DPPH-UHPLC-UV methods revealed very weak antioxidant activity of cyclitols, comparing with those of flavanones and glutathione. The major novelty of the present studies is the discovery of synergy between glutathione by methyl-ether derivatives of cyclitols (d-pinitol and l-quebrachitol), leading to significant enhancement of the antioxidative properties of their mixtures. It can be expected that this information will serve clinicians to study how the amount of glutathione produced by the body or its supplementation affects the efficacy of cyclitols as drugs in several diseases. Moreover, a weaker synergism between glutathione and some flavanones enables us to the suggestion that mixtures of glutathione, cyclitols, and some flavanones could increase their antioxidative properties/therapeutic effects.

## Figures and Tables

**Table 1:** Antioxidant activity of mixtures of cyclitols with flavonoids and glutathione.

Cyclitol	RSA%	Antioxidant	RSA%	Theoretical RSA%	Experimental RSA%	Change of RSA%
d-Pinitol (10 mg/mL)	19.3	Hesperetin (50 μg/mL)	43.6	62.9	36.4	**↓↓**
		Naringenin (100 μg/mL)	25.8	45.1	21.2	**↓↓**
		Eriodictyol (2 μg/mL)	41.9	61.2	37.0	**↓↓**
		Liquiritigenin (20 μg/mL)	22.6	41.9	21.5	**↓↓**
		Glutathione (10 μg/mL)	30.7	50.0	54.0	**↔**
d-Pinitol from carob (10 mg/mL)	6.6	Hesperetin (50 μg/mL)	43.6	50.2	37.9	**↓↓**
		Naringenin (100 μg/mL)	25.8	32.4	16.9	**↓↓**
		Eriodictyol (2 μg/mL)	41.9	48.5	34.9	**↓**
		Liquiritigenin (20 μg/mL)	22.6	29.2	21.6	**↔**
		Glutathione (10 μg/mL)	30.7	37.3	79.9	**↑↑**
l-Quebrachitol (10 mg/mL)	14.0	Hesperetin (50 μg/mL)	43.6	57.6	58.1	**↔**
		Naringenin (100 μg/mL)	25.8	39.8	18.1	**↓↓**
		Eriodictyol (2 μg/mL)	41.9	55.9	34.1	**↓↓**
		Liquiritigenin (20 μg/mL)	22.6	36.6	20.5	**↓**
		Glutathione (10 μg/mL)	30.7	44.7	42.7	**↔**
l-*chiro*-Inositol (10 mg/mL)	9.8	Hesperetin (50 μg/mL)	43.6	53.7	38.8	**↓**
		Naringenin (100 μg/mL)	25.8	35.6	20.2	**↓**
		Eriodictyol (2 μg/mL)	41.9	51.7	31.3	**↓↓**
		Liquiritigenin (20 μg/mL)	22.6	32.4	16.2	**↓↓**
		Glutathione (10 μg/mL)	30.7	40.5	53.9	**↑↑**
d-*chiro*-Inositol (10 mg/mL)	18.5	Hesperetin (50 μg/mL)	43.6	62.1	44.2	**↓**
		Naringenin (100 μg/mL)	25.8	44.3	12.9	**↓↓**
		Eriodictyol (2 μg/mL)	41.9	60.4	36.3	**↓↓**
		Liquiritigenin (20 μg/mL)	22.6	41.1	22.2	**↓↓**
		Glutathione (10 μg/mL)	30.7	49.2	75.6	**↑↑**
*myo*-Inositol (10 mg/mL)	16.5	Hesperetin (50 μg/mL)	43.6	60.1	42.1	**↓**
		Naringenin (100 μg/mL)	25.8	42.3	24.3	**↓↓**
		Eriodictyol (2 μg/mL)	41.9	58.4	25.8	**↓↓**
		Liquiritigenin (20 μg/mL)	22.6	39.1	13.4	**↓↓**
		Glutathione (10 μg/mL)	30.7	47.2	31.8	**↓**

legend: **↓↓**—strong reduction of the antioxidant activity of the mixture; **↓**—weak decrease of the antioxidant activity of the mixture; **↑↑**—strong increase in the antioxidant activity of the mixture; **↔**—lack of change in antioxidant activity of the mixture.

**Table 2:** The IC_50_ values for selected cyclitols, flavanones, and glutathione.

Compound	IC_50_	Scavenging Activity
*Myo*-Inositol	117.3 mg/mL	very weak
d-Pinitol	14.3 mg/mL	very weak
Eriodictyol	2.8 μg/mL	very strong
Hesperetin	30.8 μg/mL	strong
Naringenin	210.0 μg/mL	weak

## Data Availability

Not applicable.

## Supplementary Materials

The following are available online. Figure S1: Chemical structure of analyzed cyclitols and methylated derivatives (l-quebrachitol and d-pinitol). Figure S2. Changes in absorbance of l-quebrachitol, flavanones, and glutathione and mixtures of l-quebrachitol with flavanones and glutathione. Figure S3: The GC-FID chromatograms of TMS-derivatives of d-pinitol: from Sigma (A) and isolated from carob (B). Abbreviations: 1—internal standard (xylitol), 2—d-pinitol; 3—d-*chiro*-inositol, 4—*myo*-inositol. Figure S4a: The comparison of mass spectra of d-pinitol purified from carob (A, compound no 2 on Figure S3B) with original standard (B) and d-pinitol from NIST Library (C). Figure S4b. The comparison of mass spectra of d-*chiro*-inositol (A, compound no 3 on Figure S3B), found as an impurity of d-pinitol isolated from carob, with mass spectra of original standard of d-*chiro*-inositol (B) and d-*chiro*-inositol from NIST Library (C). Figure S4c: The comparison of mass spectra of *myo*-inositol (A, compound no 4 on Figure S3B), found as impurity of d-pinitol isolated from carob, with mass spectra of original standard of *myo*-inositol (B) and *myo*-inositol from NIST Library (C).

## Sample Availability

Samples of d-pinitol purified from carob are available from the authors.

## Abbreviations

CUPRAC	Cupric Ion Reducing Antioxidant Capacity
DPPH	2,2-diphenyl-1-picrylhydrazyl radical
UHPLC-UV	ultra-high performance liquid chromatography with ultraviolet detection
RSA	radical scavenging activity
IC_50_	50% inhibition of the radical scavenging effect
GC-FID	high-resolution gas chromatography with flame ionization detection
GC-MS	gas chromatography with mass spectrometry detection
