# Supplementary material for: Analyses of Antioxidative Properties of Selected Cyclitols and Their Mixtures with Flavanones and Glutathione
Source: Molecules. 2021 Dec 28;27(1):158. doi: 10.3390/molecules27010158 (PMC8746988; doi:10.3390/molecules27010158)
Supplement: Supplementary file 1 [file molecules-27-00158-s001.zip › molecules-1521737-supplementary.pdf]

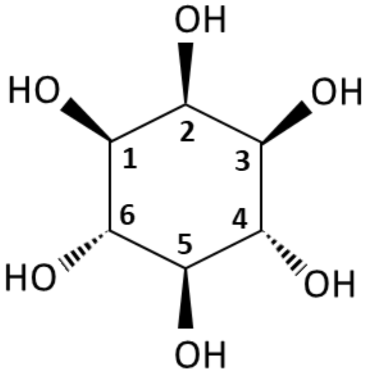

myo-Inositol

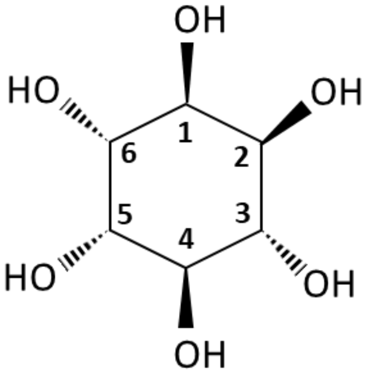

1L-*chiro*-Inositol

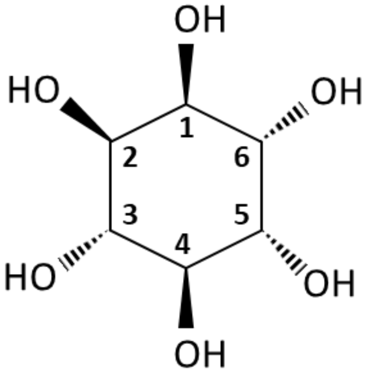

1D-*chiro*-Inositol

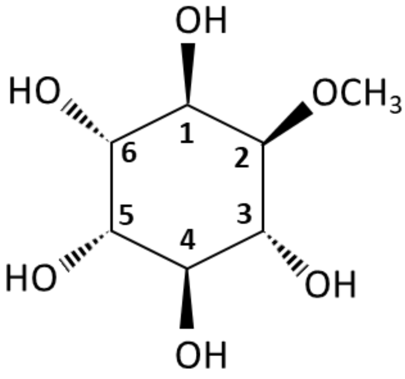

L-Quebrachitol
(1L-2-*O*-methyl-*chiro*-inositol)

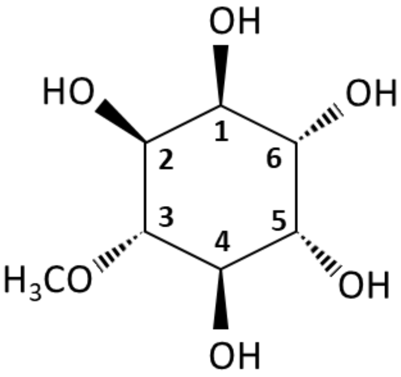

D-Pinitol
(3-*O*-D-*chiro*-inositol)

Figure S1. Chemical structure of analyzed cyclitols and their methylated derivatives (L-quebrachitol and D-pinitol)

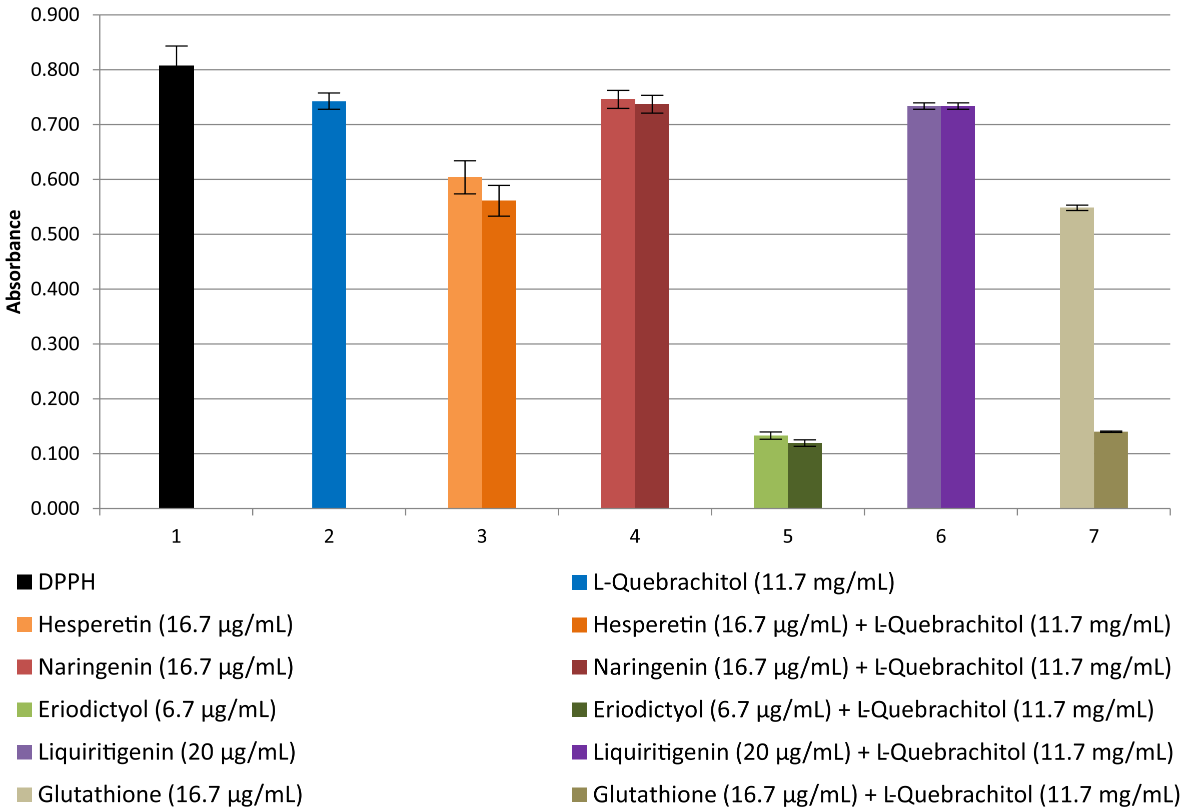

Figure S2. Changes in absorbance of L-quebrachitol, flavanones and glutathione and mixtures of L-quebrachitol with flavanones and glutathione

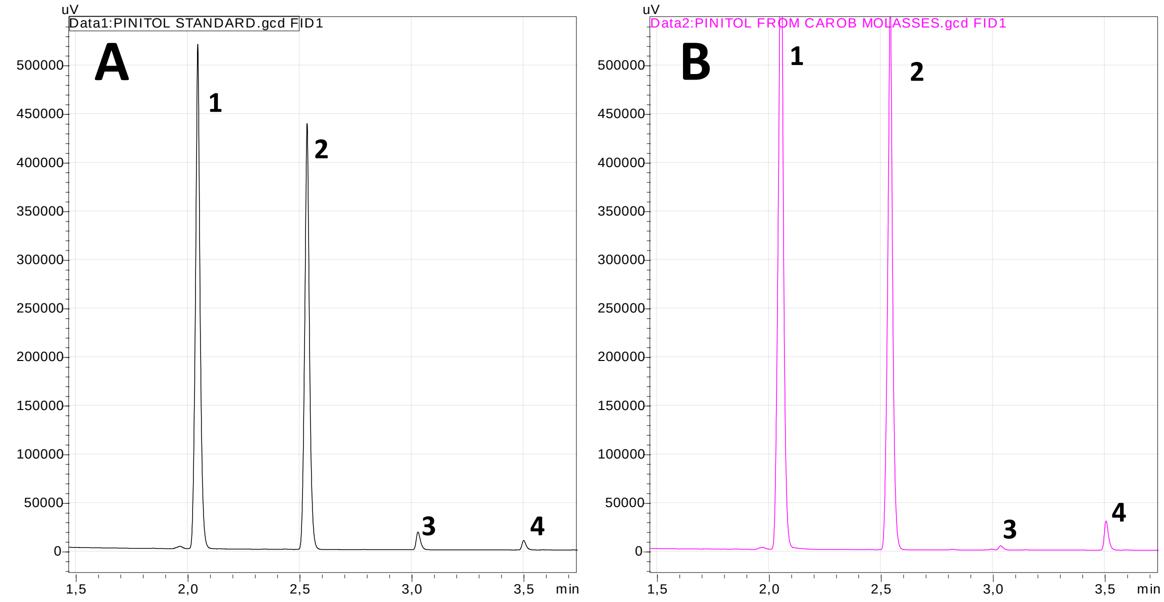

Figure S3. The GC-FID chromatograms of TMS-derivatives of D-pinitol: from Sigma (**A**) and isolated from carob (**B**). Abbreviations: 1 – internal standard (xylitol), 2 – D-pinitol; 3 – D-*chiro*-inositol, 4 – *myo*-inositol

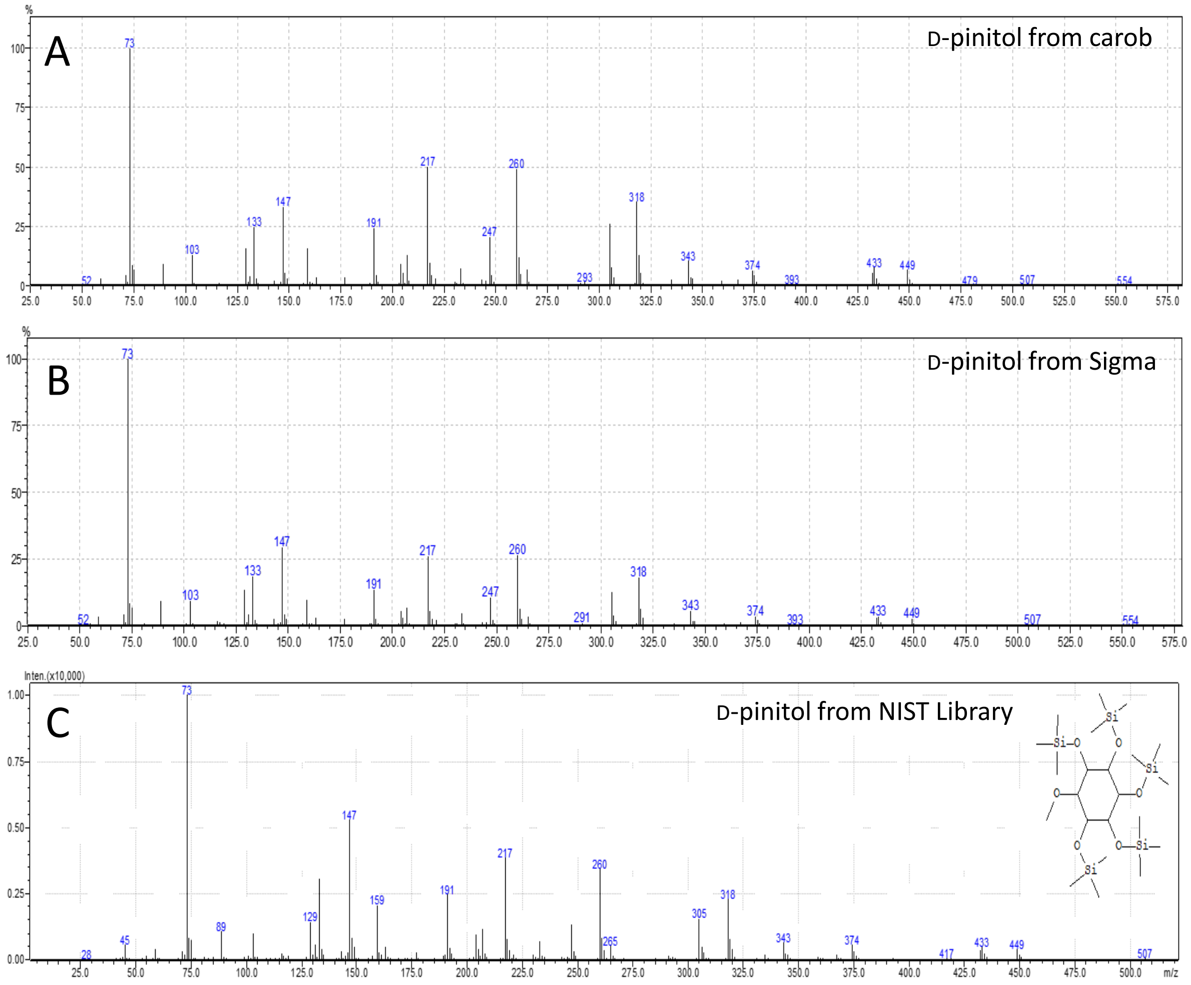

Figure S4a. The comparison of mass spectra of D-pinitol purified from carob (**A**, compound no 2 on Fig. S3B) with mass spectra of original standard of D-pinitol (**B**) and D-pinitol from NIST Library (**C**).

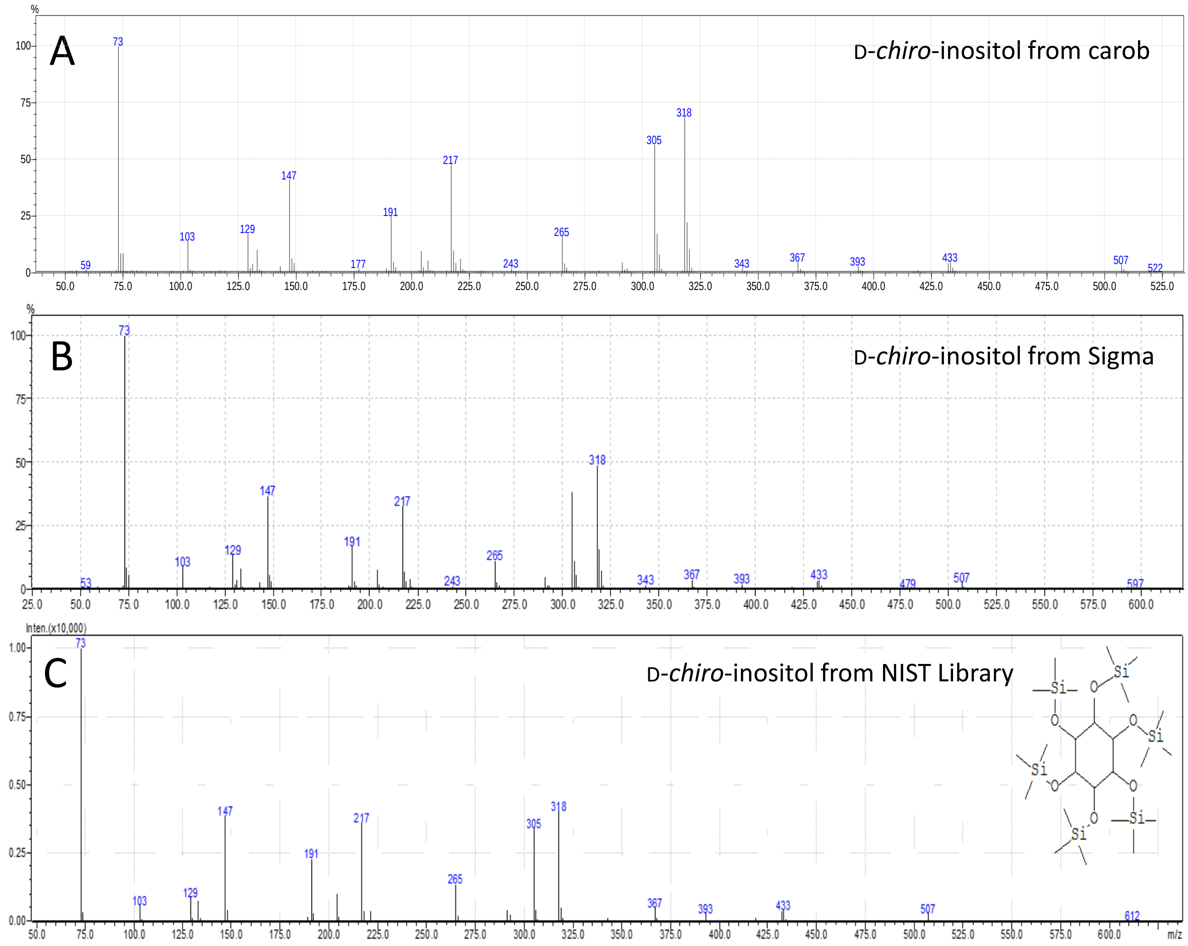

Figure S4b. The comparison of mass spectra of D-*chiro*-inositol (**A**, compound no 3 on Fig. S3B), found as impurity of D-pinitol isolated from carob, with mass spectra of original standard of D-*chiro*-inositol (**B**) and D-*chiro*-inositol from NIST Library (**C**).

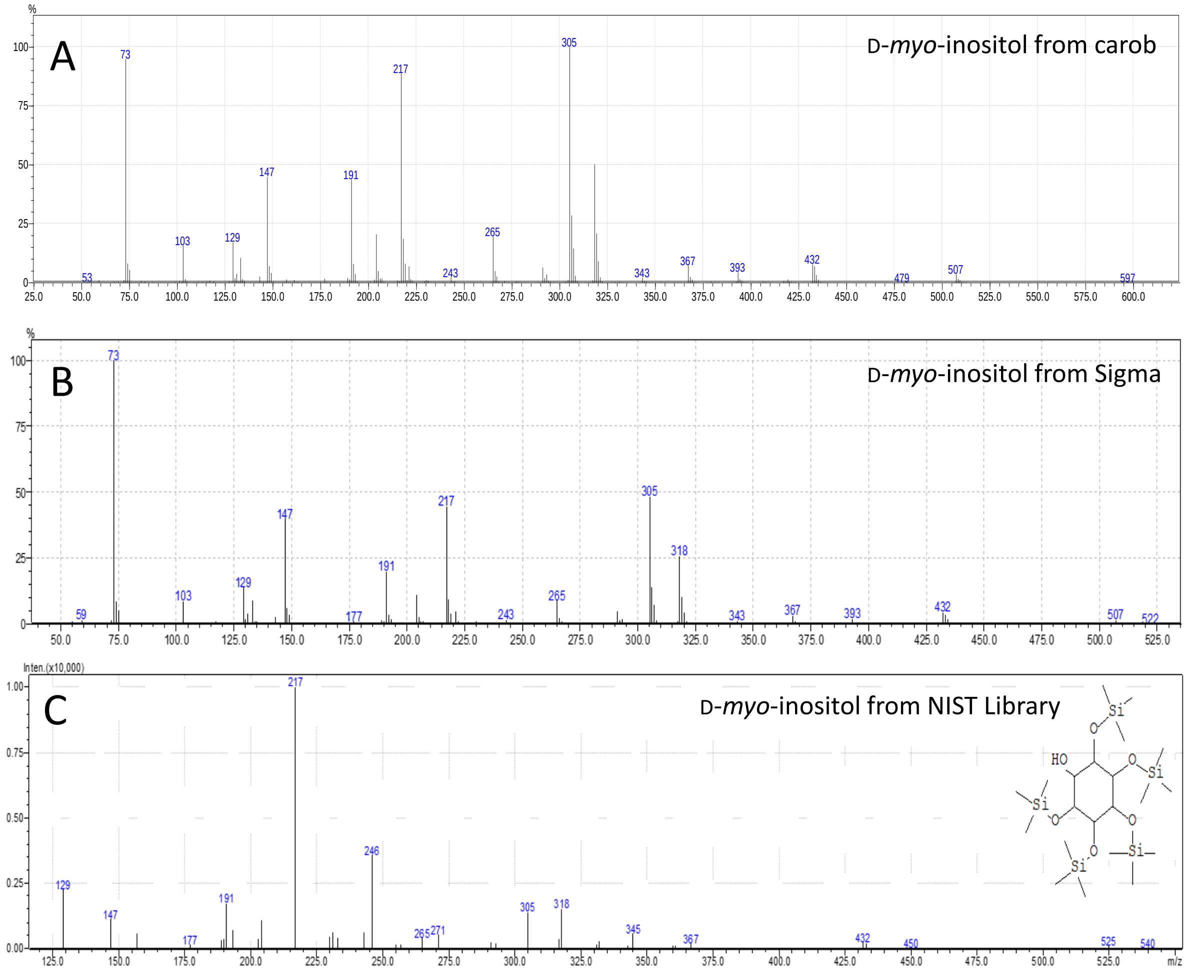

Figure S4c. The comparison of mass spectra of *myo*-inositol (**A**, compound no 4 on Fig. S3B), found as impurity of D-pinitol isolated from carob, with mass spectra of original standard of *myo*-inositol (**B**) and *myo*-inositol from NIST Library (**C**).
